# Application of Polymerization Activator in the Course of Synthesis of *N*-Isopropylacrylamide Derivatives for Thermally Triggered Release of Naproxen Sodium

**DOI:** 10.3390/ma11020261

**Published:** 2018-02-08

**Authors:** Monika Gasztych, Anna Kotowska, Witold Musiał

**Affiliations:** Department of Physical Chemistry, Faculty of Pharmacy, Wroclaw Medical University, 50-556 Wroclaw, Poland; monika.gasztych@umed.wroc.pl (M.G.); anna.kotowska1@gmail.com (A.K.)

**Keywords:** *N*-isopropylacrylamide, polymer synthesis, thermosensitive hydrogel, drug release, naproxen sodium

## Abstract

Poly-*N*-isopropylacrylamide (polyNIPA) is an extensively studied polymer in the field of controlled drug delivery. PolyNIPA contains carbonyl and amide groups along a hydrophobic chain. In an aqueous environment, crosslinked polyNIPA forms a gel characterized by a reversible volume phase transition temperature (VPTT), in response to changes in the external environment excited by the temperature factor. NIPA-based polymers were synthesized by a surfactant-free precipitation polymerization (SFPP) method at a temperature of 70 °C using the free radical initiator potassium persulfate (KPS) and at 35 °C using redox initiator system KPS with *N*,*N*,*N*’,*N*’-tetramethylethylenediamine (TEMED). The synthesized products were evaluated via dynamic light scattering (DLS), nuclear magnetic resonance (NMR) and Fourier-transform infrared spectroscopy (FTIR). The chemical structure, molecular mass, and hydrodynamic diameter of obtained particles, as well as the effects of synthesized polymers on the release of the active substance, naproxen sodium (NS), from hydroxypropyl methyl cellulose (HPMC)-based hydrogels were assessed. The use of the TEMED activator affected the particle size, as well as the release kinetics of NS. The insertion of TEMED into reactant mixtures may be applied to modify the release kinetics of NS from hydrogel preparations.

## 1. Introduction

Hydrogels are three-dimensional crosslinked water-soluble networks. The high water content in hydrogels results in good biocompatibility of the systems prepared on the basis of the hydrophilic polymers. Hydrogels were first used to make contact lenses; however later they were applied as controlled drug delivery systems. Initially, hydrogels were designed to protect the drug molecule against adverse environmental conditions or to form a depot preparation, slowly releasing the drug substance. The intensive development of polymer chemistry has gone further in the synthetic applications of hydrogels and has led to the emergence of “intelligent polymers” that react to several external stimuli [1,2,3,4]. Crosslinked intelligent polymers are used in a variety of materials, depending on the sensitivity factors. Hydrophilic gels have been known to change their properties in response to temperature changes, pH, light intensity, mechanical pressure or electrical impulses [5,6,7]. The discovery of intelligent polymers has opened new possibilities for drug controlled delivery systems. The biggest challenge in designing new structures is their rapid response to external stimuli. The parameters that still need improvement are the mechanical durability, biocompatibility, and for internal use, biodegradability [8,9,10]. Thermosensitive, water-soluble polymers have their specific properties because of the presence of hydrophobic mostly methyl, ethyl, and propyl groups. The swelling of most of the compounds in this group increases with the increase in temperature and is described by the upper critical solution temperature (UCST), including, for example, polyacrylic acid and polyacrylamide combined with butyl methacrylate. In the course of swelling, they lose their structure and release the active substance from the internal part of the polymeric network [11,12]. Poly-*N*-isopropylacrylamide (polyNIPA) is an extensively studied polymer in the field of controlled drug delivery, because of its biocompatibility and an interesting volume phase transition temperature (VPTT), which is close to body temperature (ca. 33 °C). Non-crosslinked polyNIPA is water soluble in any ratio at low temperatures; however an increased temperature results in phase separation and precipitation of the polymer. On the other hand, the crosslinked polymer rapidly shrinks at a lower critical solution temperature (LCST). These polyNIPA properties are related to the presence of the isopropyl groups in the side chain [13,14,15]. The application of various comonomers and chemical substrates may lead to the modification of the LCST of synthesized polyNIPA. Reduction in the VPTT may be achieved by the insertion of hydrophobic groups, for example, butyl methacrylate. Oppositely, an increase in the LCST may be evoked in the presence of additional hydrophilic groups, that is, by acrylamide groups. The LCST depends on the degree of ionization and thus also on the pH, when ionizable groups are present in the polymeric network, for example, carboxyl groups. In this case, small pH changes can lead to significant differences in volume at a constant temperature [16,17,18]. The use of NIPA derivatives may be beneficial as a result of supposed photoprotective properties of these compounds, according to the ability of polymeric structures to absorb UV radiation [19]. The collapse of the polyNIPA network, along with the temperature increase, depends also on the pH value. Modification of the hydrogel structure influences the pH sensitivity of the described thermosensitive hydrogels [20,21,22].

The aim of this work is the evaluation of the effect of the polymerization activator *N*,*N*,*N*’,*N*’-tetramethylethylenediamine (TEMED) on the selected physical and chemical properties of NIPA derivatives and on the release of naproxen sodium (NS) from hydrophilic gels with synthesized NIPA derivatives.

The list of applied acronyms is the following: BF—best fit; DLS—dynamic light scattering; DMSO—dimethylsulfoxide; FO—first order; FTIR—Fourier-transform infrared spectroscopy; H—Higuchi; HPMC—hydroxypropyl methyl cellulose; K—release rate; KPS—potassium persulfate; LCST—lower critical solution temperature; MBA—*N*,*N*’-methylene bisacrylamide; MWCO—molecular weight cut-off; M_w_—molecular weight; NIPA—*N*-isopropylacrylamide; NMR—nuclear magnetic resonance; NS—naproxen sodium; NTB—*N*-tertbutyl acrylamide; PEG-DMA—poly(ethylene glycol) dimethacrylate; polyNIPA—poly-*N*-isopropylacrylamide; REF—reference gel; S—second order; SD—standard deviation; SEM—scanning electron microscopy; SFPP—surfactant-free precipitation polymerization; SLS—static light scattering; TEMED—*N*,*N*,*N*’,*N*’-tetramethylethylenediamine; UCST—upper critical solution temperature; USP—United States Pharmacopoeia; VPTT—volume phase transition temperature; ZO—zero-order process.

## 2. Results

### 2.1. Nuclear Magnetic Resonance Spectroscopy

**Figure 1:**
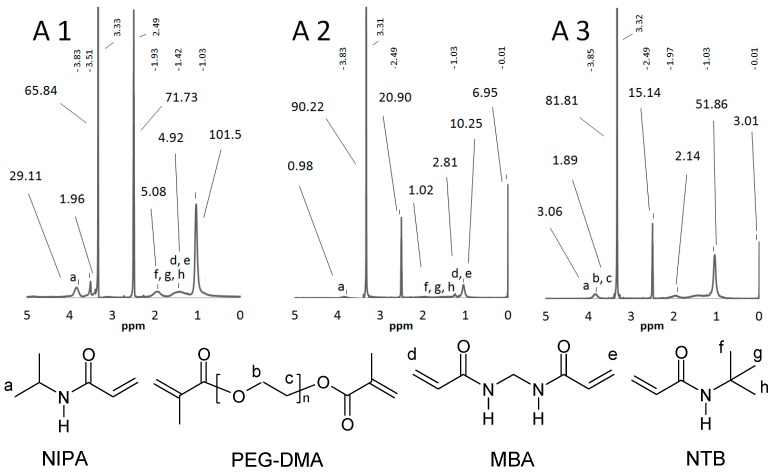
^1^H-NMR spectra of A1–A3. The shifts (at the **top** of the spectra, small digits) and respective integrals (below the shifts, bigger digits) are presented above the spectral line.

The chemical structures of the synthesized polymers, compared to the applied components, were evaluated with NMR spectroscopy. The ^1^H-NMR spectra of the products are presented in Figure 1. The NMR spectra of the NIPA monomer possess characteristic multiplets in the region δ: 5.5 ppm–6.4 ppm (vinyl protons). These signals are absent in the A1–A3 spectra because of the polymerization process and the disappearance of monomers and oligomers in the products. The signals in the region δ: 3.85 ppm evidences the inherency of tertiary protons of the isopropyl group. The broad signals at δ: 1.25 ppm confirm the presence of protons of the methylene group (MBA). The presence of PEG-DMA is elucidated by a characteristic multiplet of ethylene groups (δ: 3.4 ppm–3.9 ppm). Signals at δ: 1.32 ppm for polymer A2 support the presence of a methylene group of NTB.

### 2.2. Fourier-Transform Infrared Spectroscopy

**Figure 2:**
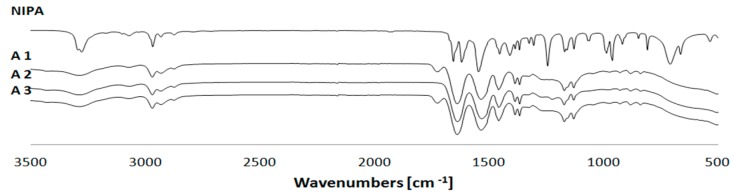
Fourier-transform infrared spectroscopy (FTIR) spectra of obtained A1–A3 particles and main monomer *N*-isopropylacrylamide (NIPA). The disappearance of vinyl bonds signals (3104, 3030, 1620, and 1409 cm^−1^) confirms the insertion of the monomer into the polymer particle.

SFPP results in a saturation vinyl bond, which is observed in the FTIR as a disappearance of characteristic frequencies above 3000 cm^−1^. In fact, the maxima at 3104 and 3030 cm^−1^ reflecting the stretching vibrations of the saturated double bond between carbon atoms were absent on the polymer spectrum. The frequency bands of 1620 and 1409 cm^−1^ also vanished, as a result of the completion of the synthesis (Figure 2).

### 2.3. Hydrodynamic Diameter and Volume Phase Transition Temperature

The hydrodynamic diameter (D_H_) at 18 and 42 °C and VPTT are presented in Table 1. Polymers synthesized with the activator TEMED at low temperatures (A2 and A3) exhibited high VPTT values. The A1 product exhibited the lowest value of D_H_ at 18 °C, whereas at 42 °C, the A2 product gave the smallest diameter.

### 2.4. Approximation of Molecular Mass of Synthesized Polymers

The molecular weight (M_w_) of the polymers was assessed at 25 °C. The results are presented in Table 2. There are noticeable differences in the results between the synthesized polymers, which may be result of the use of TEMED and varied comonomers, as well as crosslinkers. Extreme differences in the M_w_ values between the particles synthesized with TEMED were observed. A2 particles presented the lowest M_w_ values of around 27.73 kDa. The insertion of PEG-DMA to the A3 polymer resulted in a high increase in the value of M_w_ up to 3430.00 kDa.

### 2.5. Morphology of the Polymers Measured by Scanning Electron Microscopy

**Figure 3:**
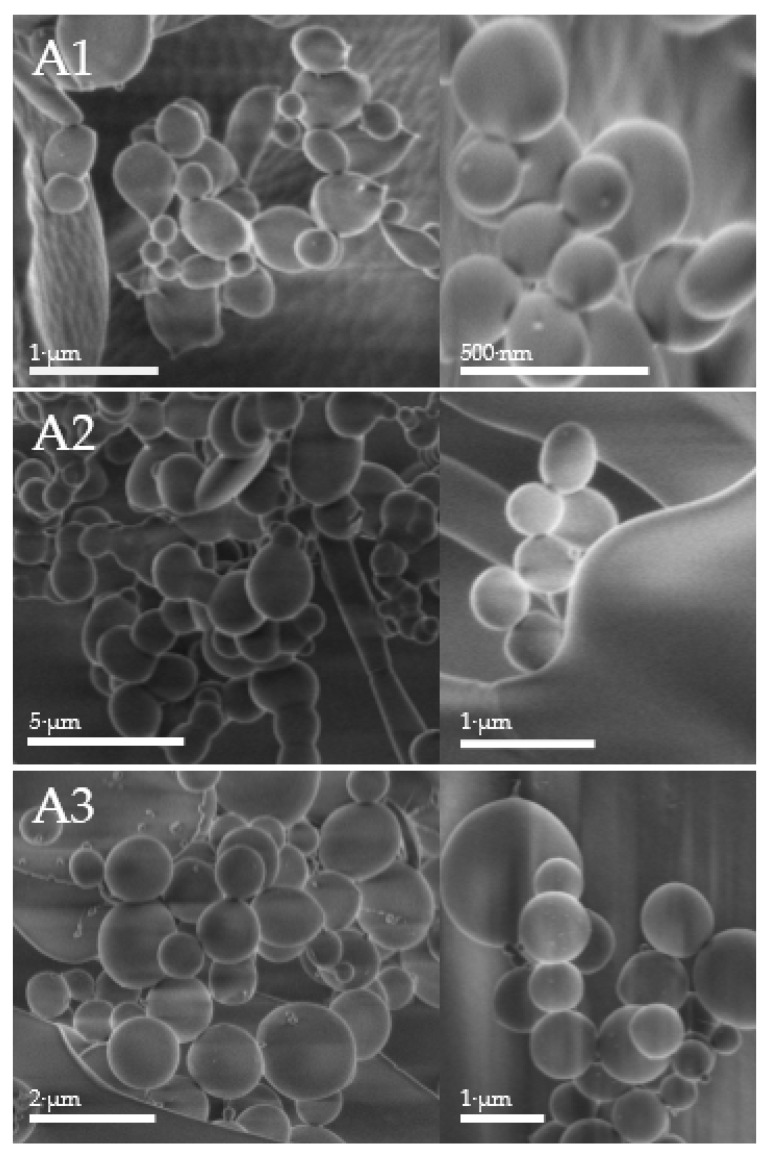
Scanning electron microscopy (SEM) of obtained particles A1–A3. The diameters measured under the SEM vacuum conditions were in the same range for the evaluated polymers A1–A3.

The obtained SEM photographs indicate the inhomogeneity of the synthesized particles; however, it can be observed that most of the A1 particles obtained during the first synthesis were in the range of 0.5 μm–1.0 μm. As shown in Figure 3, there were also groups of very small spheres of around 200 nm. In the SEM photographs of the A2 particles, it can be noticed that the microspheres evaluated with TEMED presented larger diameters than the A1 particles. A2 and A3 particles were larger than those obtained during the first A1 synthesis. The size reduction may have been a result of the use of another crosslinking agent or the lack of additional comonomers in the product structure.

### 2.6. Release Kinetics of Naproxen Sodium from Thermosensitive Hydrogels

**Figure 4:**
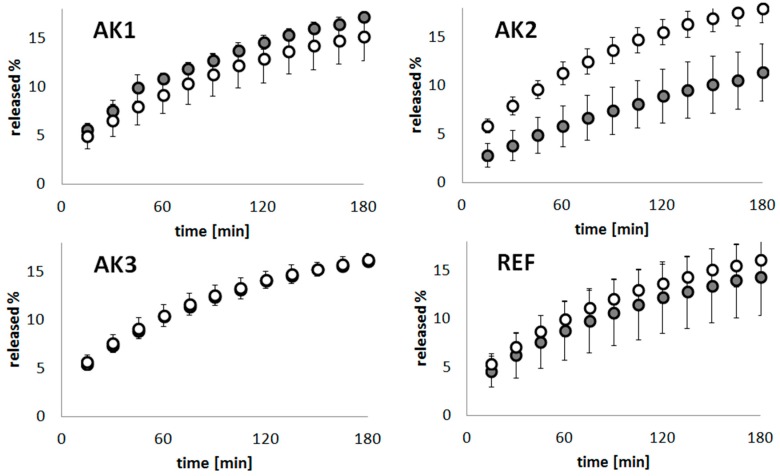
Release profiles of naproxen sodium (NS), at 22 °C (filled circle) and at 42 °C (empty circle) from the formulations AK1, AK2, AK3 and from the reference gel (REF); the Y-bars represent standard deviation.

The release kinetics were evaluated at two temperatures: 22 ± 0.5 °C and 42 ± 0.5 °C (Figure 4). A high NS quantity was released in both temperatures in the case of the reference formulation. Low NS concentrations were noted for AK2 at 22 ± 0.5 °C, while the AK1 hydrogel presented the greatest amount of released substance from the thermosensitive formulation.

Detailed calculations concerning the release rates for the selected kinetics models at various temperatures—formulations AK1–AK3 and reference gel (REF)—are presented in Table 3 and Table 4. The determination coefficients are demonstrated for the evaluated kinetics models. The Higuchi model was found to best fit the acquired data.

## 3. Discussion

**Figure 5:**
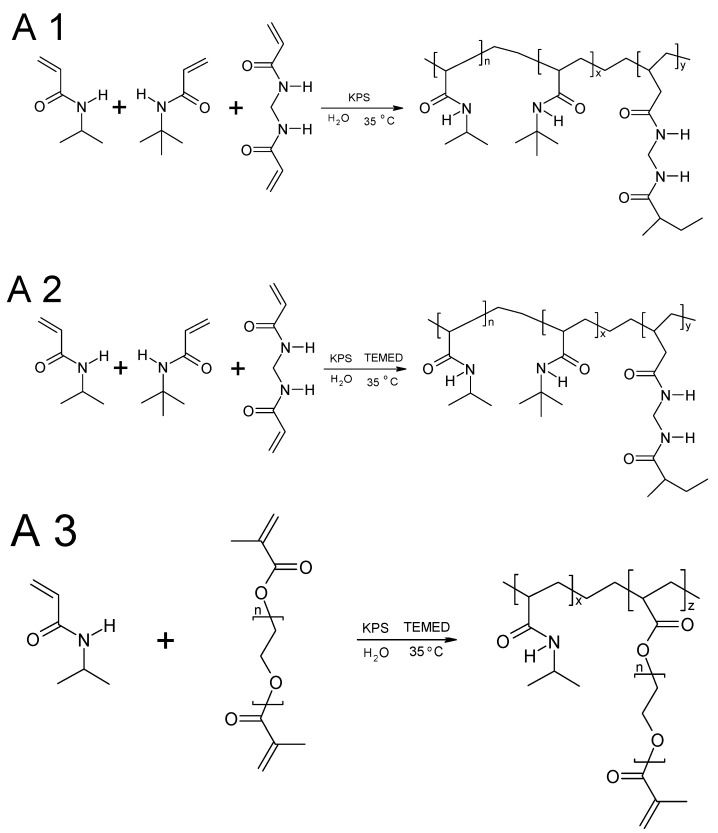
Proposed synthesis scheme of thermosensitive particles A1–A3. A1, A2 and A3 were synthesized at 35 °C; however the polymerization was activated by *N,N,N’,N’*-tetramethylethylenediamine (TEMED) in the case of A2 and A3. A3 was crosslinked by poly(ethylene glycol) dimethacrylate (PEG-DMA) instead of *N*,*N*’-methylene bisacrylamide (MBA) applied in A1 and A2.

The potential use of thermosensitive polymers in medicine has been ongoing for more than 60 years, and nowadays, the biggest challenge is to obtain a quick reversible response to external stimuli [23]. For this purpose, the synthesis conditions and used materials are constantly being optimized. Particles of different sizes and therefore various physicochemical properties were obtained in the SFPP synthesis. The proposed synthesis scheme is given on Figure 5.

The release profiles obtained in former experiments had a growing pattern similar to the logarithmic graph, similarly to the former experiments [24]. The lowest degree of release at 22 ± 0.5 °C of the active substance was found in AK2, and it may have been ascribed to the type of used crosslinker and comonomer. AK2 and AK3 released high quantities of NS at increased temperatures; however, the pronounced differences were observed in the release profile of the sample AK2. Oppositely, AK3 at an increased temperature presented a slight reduction in released quantities of NS, presumably according to the modified network structure, compared to AK1 and AK2. Interestingly, for the AK1 hydrogel at 42 ± 0.5 °C, we observed rather lower amounts of released NS, compared to at the temperature of 22 ± 0.5 °C. The crosslinking level of synthesized particles influences the rate of drug release. This may be ascribed to the contraction of the surface of the particles. The MBA crosslinked polymers exhibited higher levels of NS, released at higher temperatures compared to the PEG-DMA crosslinked formulation. The best-fitting mathematical model in all the samples was the Higuchi model, and good determination coefficients were acquired for this model [25,26]. However, there are other models applicable for the description of the performed release experiments [27]. In Table 3 and Table 4 are presented the results of a linear regression analysis performed according to selected kinetic models. The use of an activator of an initiator in the course of the synthesis affects the particles’ size. Synthesized at lower temperatures, particles with TEMED were larger in comparison to those that were synthesized without any activator, for example, the D_H_ values of A1 particles at 18 °C were around 580 nm. In the subsequent synthesis, A2 particles of relatively high D_H_ values around 860 nm were obtained. The A2 polymer had the same composition as the A1 polymer, but the TEMED activator was used. On the other hand, at the higher temperature of 42 °C, rather small values of around 130 nm for A2 and around 230 nm for A3 were noted. The high temperature contributed to the faster integration of monomers into aggregates, and the resulting particles were smaller than those that formed at the lower temperature. It may be proposed that the drug release from the AK2 formulation at 22 °C is slower, as a result of the large diameter of the A2 particles at 18 °C, compared to AK1. The differences between samples, in terms of size and release rate, decreased, after exceeding the level of the VPTT at 42 °C.

The M_w_ values of the synthesized polymers were evaluated at a temperature of 25 °C. The results are presented in Table 2. There were clear differences between M_w_ values of the particles, approximated via SLS. Comparing the A1 and A2 polymers, the obtained results for the particles synthesized with TEMED were significantly lower and ranged around 28 kDa. The differences confirmed the strong influence of the activator on the M_w_ values of the obtained polymers. A relatively large value of around 3430 kDa was presented by A3, which may have been a consequence of the applied crosslinker PEG-DMA and the possible intercalation of the molecules. Despite many years of experiments on the intelligent polymers and the tremendous progress of production, the ideal material has not yet been found. The study confirms the thermosensitivity of NIPA derivative structures and their prospective use for drug delivery triggered by the thermal factor.

**Figure 6:**
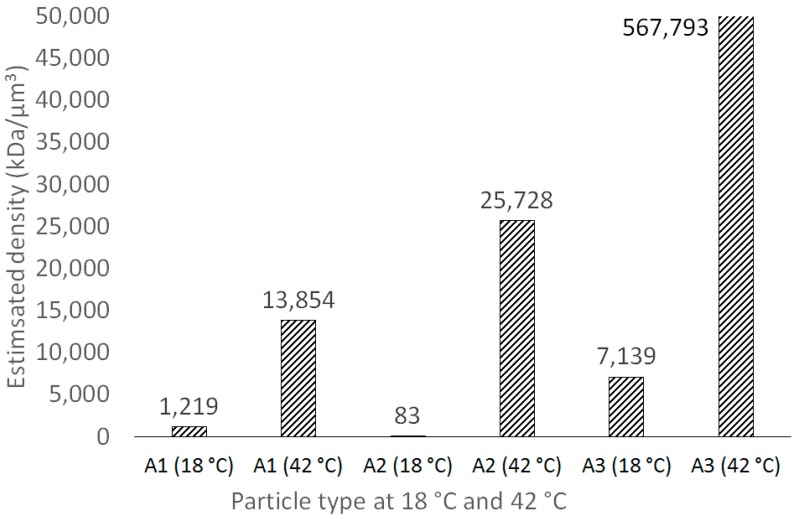
The variability of estimated density of thermosensitive particles A1–A3 at 18 and 42 °C. The highest density increase was observed for A2 (3.13∙× 10^4^%), whereas for A1 and A2, the increases were lower (1.13∙× 10^3^% and 7.96∙×10^3^%, respectively).

Interestingly, the molecular weight of the A2 polymer was relatively low, as compared to A1 and A3, whereas the D_H_ values were in the same range. This may suggest the presence of relatively high space, available for the aqueous solution of the model drug in the case of A2. When the temperature increased, the collapse of A2 particles was most prominent, compared to the A1 and A3 particles. This was confirmed by the estimation of the approximate density of the obtained macroparticles expressed as the ratio of the M_w_ value to the calculated volume of the particles (Figure 6.

The highest density increase between 18 and 42 °C was observed for A2. The phenomenon may be ascribed to the level of the crosslinking agent implemented into the macromolecule.

## 4. Experimental Section

### 4.1. Materials

NS, (*S*)-(+)-2-(6-Methoxy-2-naphthyl)propionic sodium conforming to the United States Pharmacopoeia standards (Sigma Aldrich, Steinheim, Germany), HPMC (viscosity of 2600–5600 cP, 2% in H_2_O at 20 °C; Sigma Aldrich, Ashland, Wilmington, DE, USA), NIPA (97%; Sigma Aldrich, Steinheim, Germany), NTB (99%; Acros Organics, Geel, Belgium), MBA (99%; Sigma Aldrich, Steinheim, Germany), TEMED (99%; Sigma Aldrich, Steinheim, Germany), PEG-DMA (M_w_ = 2000; Sigma Aldrich, Steinheim, Germany), and KPS (98%; BDH Laboratory Suppliers, Kampala, Uganda (GPR)) were obtained from commercial and industrial suppliers and used without further purification. The dialysis bag with a MWCO of 12.000–14.000 Da was obtained from the Visking Medicell International Ltd. Company (London, UK). Deionized water was obtained from the ionic column according to monography of the purified water from the European Pharmacopoeia, used in all studies. DMSO-d_6_ used for the NMR spectrometry was obtained from Euriso-Top (Sigma Aldrich, St Aubin, France).

### 4.2. Synthesis of the Particles

Apparatus used for the synthesis of NIPA-based polymers consisted of a 2000 mL glass reactor with a three-necked flask placed in a 4000 mL vessel as a water bath. The temperature in the reactor was constantly monitored by a temperature sensor immersed in the reacting mixture, connected to the heating system. The synthesis was carried out under an Allihn reflux condenser and under a nitrogen atmosphere. The reactor was filled with 600 mL of deionized water at a temperature of 70 °C. The redox initiator KPS was dissolved in 100 mL of deionized water and was added to the reactor flask. When the temperature was stable, the rest of the suitable components were dissolved in the deionized water and transferred to the reacting fluid. The synthesis of A1 was carried out for 6 h at 70 °C, with constant stirring, while the synthesis of A2 and A3 particles was conducted at 35 °C because of the use of the activator TEMED.

All received formulations included unreacted monomers, a crosslinking agent, an initiator and an activator (Table 5). In order to purify the products, the solutions were dialyzed after the synthesis was completed. The purification process was monitored by repeated measurements of water conductivity in the dialysis vessel every 24 h. After 144 h, the deionized water was replaced with MiliPore water (G = 0.7 μS/cm). The measurements were made on an ELMETRON CPC-511 conductivity meter with ELMETRON EC-70t electrodes. When the purification process was completed, the products were frozen and freeze-dried by Steris Lyophilizer Lyovac GT2 to use them for further experiments.

### 4.3. Nuclear Magnetic Resonance Spectroscopy

The ^1^H-NMR spectra of the used monomers and obtained particles were measured using an NMR Bruker 300 MHz spectrometer (Faellanden, Switzerland) in our facility, and measurements were recorded at a temperature of 24 °C; 5 mg of obtained polymers, previously dried, were weighed for the NMR analysis. Then they were dissolved in 0.8 mL of DMSO-D_6_. All products were soluble in DMSO; filtration and centrifugation were not required.

### 4.4. Fourier-Transform Infrared Spectroscopy

FTIR spectra of the dry polymers were found using a FTIR spectrophotometer with an attenuated total reflectance (ATR) accessory Thermo Scientific USH model Nicolet iS50 (Madison, WI, USA). This is a high-pressure switch with a monolithic diamond crystal to measure the reflected spectra. The Omnic Specta program was used for FTIR spectra analysis.

### 4.5. Hydrodynamic Diameter Measurements

The hydrodynamic diameters of the obtained polymer water dispersions were measured by a DLS method using a Zeta Sizer Nano device from Malvern Instruments (Malvern Instruments, Malvern, UK) at a wavelength of 678 nm. The dispersions of polymers were diluted 10-fold with deionized water, filtered by the polyvinylidene fluoride (PVDF) Whatmann nanofilter (0.2 μm). A polystyrene cuvette was used; it was inserted into the device, assessed in a 173° backscatter measurement arrangement and then evaluated by the Mark-Houwink parameter setting. Every measurement was repeated five times and then processed by Zetasizer Nano Software version 5.03 (Malvern Instruments Ltd, Malvern, United Kingdom).

### 4.6. Scanning Electron Microscopy

The purified and lyophilized polymer suspensions were placed on a silicon plate and then inserted into the scanning electron microscope. All measurements were taken at room temperature. The photos were obtained from the Faculty of Electronics of Microsystems and Photonics at the Wrocław University of Science and Technology.

### 4.7. Preparation Hydrogels with Naproxen Sodium

Hydrogel formulations AK1–AK3 with NS were prepared in a specific way ex tempore; 0.4 g of NS was carefully weighed, then scraped over the surface of the weighed deionized water and stirred; 0.5 g of the synthesized polymers was added after dissolving NS, then 0.5 g of HPMC was added to the solution and the mixture was made up with deionized water to 10 g. The composition of the obtained hydrogels is shown in Table 6. The whole mixture was thoroughly mixed and homogenized. The samples were incubated at a temperature of 8 °C over 24 h and were then used for the release studies at varied temperatures.

### 4.8. Evaluation of Naproxen Sodium Release Kinetics

An in vitro drug release kinetics study of NS from the thermosensitive hydrogels was performed using Erweka equipment (Erweka, Heusenstamm, Germany) with the modified pharmacopoeial paddle dissolution method at a rotation speed of around 50 rpm in 1000 mL of double-distilled water. The temperature of the process was controlled between 22 ± 0.5 °C and 42 ± 0.5 °C. Each test was executed using three samples for each gel composition (AK1, AK2 and AK3), one in each dissolution vessel; 3.0 mL of liquid was taken at every 15 min interval over 3 h. The concentration of released substance was determined spectrophotometrically using the Jasco V-530 spectrophotometer (Jasco, Tokyo, Japan) at a wavelength of *λ* = 298 nm.

### 4.9. Evaluation of Molecular Weight via Static Light Scattering

Zetasizer Nano was used to measure M_w_ values by SLS. Ten samples of increasing concentration of synthesized polymers were made to evaluate the M_w_ of the obtained polymers. To illustrate the intensity of the light diffused by the particles in the prepared solution, the Rayleigh equation was used. The M_w_ values of the polymer were evaluated in comparison with the scattering intensity of the standard solution.

## 5. Conclusions

Application of the TEMED activator at a temperature of 35 °C enabled the synthesis of nano-range particles, with pronounced VPTT, between 28 and 32 °C. The composition and polymerization conditions affected the molecular mass and the hydrodynamic diameter of the synthesized polymers, as well as the value of the VPTT. The release of the model nonsteroidal anti-inflammatory drug—NS from hydrogel with HPMC—was modified, compared to the reference, by the addition of synthesized particles. The release was modified explicitly in the case of the particles synthesized on the basis of NIPA, MBA, NTB, APS and TEMED at reduced process temperatures. The phenomenon may be ascribed to the level of crosslinking agent implemented into the macromolecule and the subsequent pattern of the shrinking of the particles. The use of PEG-DMA influenced slightly the release process of NS from the HPMC base.

## Figures and Tables

**Table 1:** Hydrodynamic diameter D_H_ and volume phase transition temperature (VPTT) values at 18 and 42 °C temperatures of the synthesized polymers.

Type of Polymer	D_H_ at 18 °C (nm)	SD	D_H_ at 42 °C (nm)	SD	VPTT (°C)
A1	577.00	3.25	256.6	3.86	28
A2	861.47	17.2	127.23	1.1	30
A3	971.93	30.79	226.37	5.8	28–32

A1–A3: synthesized polymers via surfactant-free precipitation polymerization (SFPP); SD—standard deviation.

**Table 2:** Molecular weight (M_w_) of synthesized polymers measured via static light scattering (SLS); SD—standard deviation.

Type of Polymer	Average M_W_ (kDa)	SD
A1	122.50	23.33
A2	27.73	4.74
A3	3430.00	14.14

**Table 3:** The release rates K_ZO_, K_FO_, K_SO_, K_H_, and determination coefficients (R^2^), respectively, for the evaluated kinetics models: ZO—zero-order process, FO—first-order process, SO—second-order process, and H—Higuchi model for the release of naproxen sodium (NS) from formulations AK1–AK3 and REF, at temperature of 22 °C.

Model	Parameter	Type of Formulation							
		AK1		AK2		AK3		REF	
		Value	SD	Value	SD	Value	SD	Value	SD
ZO	K_ZO_ (% min^−1^)	6.08∙× 10^−2^	5.95∙× 10^−3^	5.68∙× 10^−2^	5.31∙× 10^−3^	6.20∙× 10^−2^	1.42∙× 10^−3^	6.08∙× 10^−2^	1.84∙× 10^−2^
	r^2^	0.96485	0.00992	0.98365	0.01123	0.94852	0.00450	0.94085	0.01771
FO	K_FO_ (min^−1^)	6.82∙× 10^−4^	8.19∙× 10^−5^	6.20∙× 10^−4^	7.29∙× 10^−5^	7.01∙× 10^−4^	1.84∙× 10^−5^	6.90∙× 10^−4^	2.26∙× 10^−4^
	r^2^	0.97030	0.00848	0.98626	0.00910	0.95540	0.00416	0.94805	0.01897
SO	K_SO_ (min^−1^∙%^−1^)	7.65∙× 10^−6^	1.09∙× 10^−6^	6.78∙× 10^−6^	9.59∙× 10^−7^	7.92∙× 10^−6^	2.36∙× 10^−7^	7.85∙× 10^−6^	2.76∙× 10^−6^
	r^2^	0.97526	0.00716	0.98843	0.00698	0.96177	0.00379	0.95471	0.01989
H	K_H_ (min^0.5^)	1.11	1.13∙× 10^−1^	1.02	1.05∙× 10^−1^	1.14	2.40∙× 10^−2^	1.12	3.31∙× 10^−1^
	r^2^	0.99723	0.00154	0.99226	0.00660	0.99335	0.00129	0.99067	0.00680
BF		H		H		H		H	

SD—standard deviation; BF—best fit.

**Table 4:** The release rates K_ZO_, K_FO_, K_SO_, K_H_, and determination coefficients (R^2^), respectively, for the evaluated kinetics models: ZO—zero-order process, FO—first-order process, SO—second-order process, and H—Higuchi model for the release of naproxen sodium (NS) from formulations AK1–AK3 and REF, at temperature of 42 °C.

Model	Parameter	Type of Formulation							
		AK1		AK2		AK3		REF	
		Value	SD	Value	SD	Value	SD	Value	SD
ZO	K_ZO_ (% min^−1^)	6.73∙× 10^−2^	2.64∙× 10^−3^	7.14∙× 10^−2^	4.96∙× 10^−3^	6.12∙× 10^−2^	1.99∙× 10^−3^	5.93∙× 10^−2^	3.48∙× 10^−3^
	r^2^	0.95662	0.01412	0.95397	0.00820	0.94747	0.02971	0.96221	0.02017
FO	K_FO_ (min^−1^)	7.65∙× 10^−4^	2.46∙× 10^−5^	8.19∙× 10^−4^	7.18∙× 10^−5^	6.93∙× 10^−4^	1.37∙× 10^−5^	6.64∙× 10^−4^	5.17∙× 10^−5^
	r^2^	0.96358	0.01321	0.96133	0.00670	0.95426	0.02758	0.96784	0.01815
SO	K_SO_ (min^−1^∙%^−1^)	8.70∙× 10^−6^	2.18∙× 10^−7^	9.40∙× 10^−6^	9.97∙× 10^−7^	7.86∙× 10^−6^	5.45∙× 10^−8^	7.45∙× 10^−6^	7.23∙× 10^−7^
	r^2^	0.96986	0.01225	0.96803	0.00532	0.96056	0.02544	0.97300	0.01621
H	K_H_ (min^0.5^)	1.23	4.01∙× 10^−2^	1.30	9.48∙× 10^−2^	1.12	2.41∙× 10^−2^	1.08	7.24∙× 10^−2^
	r^2^	0.99371	0.00175	0.99455	0.00200	0.99149	0.00913	0.99638	0.00402
BF		H		H		H		H	

SD—standard deviation; BF—best fit.

**Table 5:** Substrate composition of the obtained particles.

Substrate % (*w/w*)		Type of Component					
		Main Monomer	Initiator	Activator	Crosslinker	Comonomer	Solvent
**Type of Polymer**	**A1**	NIPA	KPS		MBA	NTB	water
		0.5	0.05		0.05	0.05	99.35
	**A2**	NIPA	KPS	TEMED	MBA	NTB	water
		0.5	0.05	0.02	0.05	0.05	99.33
	**A3**	NIPA	KPS	TEMED	PEG-DMA		water
		0.5	0.05	0.02	0.05		99.38

NIPA—*N*-isopropyl acrylamide; KPS—potassium persulfate; TEMED—*N*,*N*,*N*’,*N*’-tetramethyl ethylenediamine; MBA—*N*,*N*’-methylene bisacrylamide; PEG-DMA—poly(ethylene glycol) dimethacrylate; NTB—*N*-tertbutyl acrylamide.

**Table 6:** Compositions of hydrogels AK1–AK2 with the synthesized particles and reference formulation.

Type of Hydrogel	Component (%)					
	NS	A1	A2	A3	HPMC	AQ
AK1	4	5	-	-	5	86
AK2	4	-	5	-	5	86
AK3	4	-	-	5	5	86
REF	4	-	-	-	5	91

NS—naproxen sodium; A1–A3—synthesized polymers dispersions by SFPP according to the Table 1; REF—reference gel; HPMC—hydroxypropyl methylcellulose; AQ—water.
